# Simulation Study on the Structure Design of p-GaN/AlGaN/GaN HEMT-Based Ultraviolet Phototransistors

**DOI:** 10.3390/mi13122210

**Published:** 2022-12-13

**Authors:** Haiping Wang, Haifan You, Jiangui Yang, Minqiang Yang, Lu Wang, Hong Zhao, Zili Xie, Dunjun Chen

**Affiliations:** 1 School of Electronic Science and Engineering, Nanjing University, Nanjing 210023, China; 2 Nanjing Sanchahe River Estuary Sluice Management Office, Nanjing 210098, China

**Keywords:** ultraviolet (UV), phototransistors (PTs), p-GaN/AGaN/GaN, HEMTs, Silvaco, simulation

## Abstract

This work investigates the impacts of structural parameters on the performances of p-GaN/AlGaN/GaN HEMT-based ultraviolet (UV) phototransistors (PTs) using Silvaco Atlas. The simulation results show that a larger Al content or greater thickness for the AlGaN barrier layer can induce a higher two-dimensional electron gas (2DEG) density and produce a larger photocurrent. However, they may also lead to a larger dark current due to the incomplete depletion of the GaN channel layer. The depletion conditions with various Al contents and thicknesses of the AlGaN layer are investigated in detail, and a borderline between full depletion and incomplete depletion was drawn. An optimized structure with an Al content of 0.23 and a thickness of 14 nm is achieved for UV-PT, which exhibits a high photocurrent density of 92.11 mA/mm, a low dark current density of 7.68 × 10^−10^ mA/mm, and a large photo-to-dark-current ratio of over 10^11^ at a drain voltage of 5 V. In addition, the effects of other structural parameters, such as the thickness and hole concentration of the p-GaN layer as well as the thickness of the GaN channel layer, on the performances of the UV-PTs are also studied in this work.

## 1. Introduction

Ultraviolet (UV) photodetectors based on III-nitride semiconductors have numerous applications in military and civilian fields [1]. Various types of GaN photodetectors, such as the Schottky barrier [2,3], metal-semiconductor-metal [4,5], and p-i-n [6,7], have been widely explored. However, these usually do not have optical gains, which limits the scope of their applications.

In addition to the conventional photodetector configurations, phototransistors (PTs) based on AlGaN/GaN HEMT structures [8] have also been presented. Three methods of gate control have been explored, including recessed barrier gate [9,10,11], Schottky gate [12,13,14], and p-GaN gate [15,16,17]. Among these approaches, PT with a p-GaN optical gate has shown excellent comprehensive performance. PT with a p-GaN optical gate was first reported by Iwaya et al. in 2009 [15]. The device showed a large optical gain and a relatively low dark current. In 2020, Lyu et al. analyzed the working mode of PTs and reported a device with a much lower leakage current as well as a large photo-to-dark current ratio [16]. Recently, our group demonstrated a device with a fast response time to the microsecond level while maintaining excellent electrical performances [17]. The above progress suggests that p-GaN/AGaN/GaN HEMT-based UV PT exhibits both high responsivity and fast response time and shows great potential for high-performance UV detection.

In this work, the impacts of structural parameters on the performances of p-GaN/AlGaN/GaN HEMT-based UV PTs are systematically investigated using Silvaco software. The electron distribution, polarization charge density, polarization-induced two-dimensional electron gas (2DEG) density, and conduction band diagrams are calculated to reveal the influence of hole concentration, layer thickness, and alloy composition on dark current and photocurrent. The variations of depletion conditions with the Al content and thickness of the AlGaN barrier are investigated in detail, and a borderline for full depletion and incomplete depletion was drawn as a reference of device design for subsequent researchers.

## 2. Device Structure and Simulation Models

**Figure 1:**
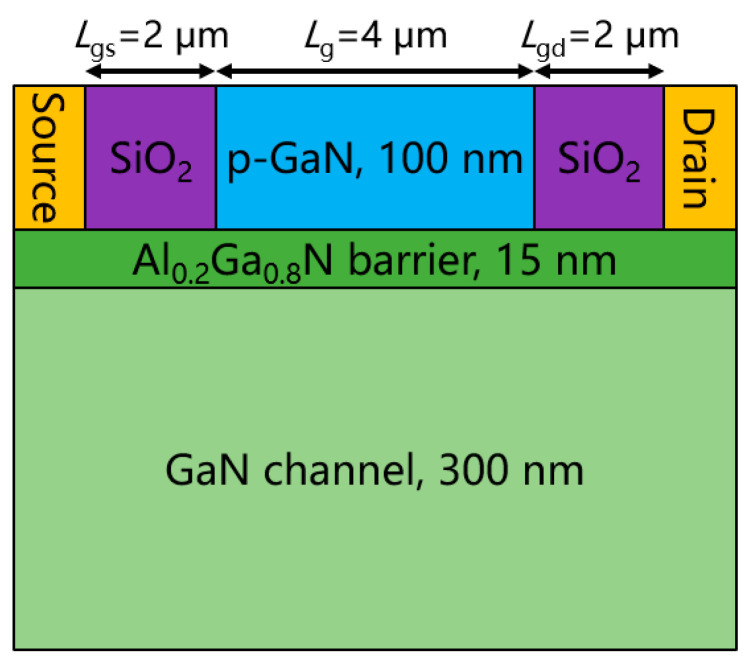
Schematic structure of a typical PT based on p-GaN/AGaN/GaN HEMTs.

Figure 1 plots the schematic structure of a typical PT, which consists of a 100-nm-thick p-GaN layer (p~1 × 10^18^ cm^−3^), a 15-nm-thick Al_0.2_Ga_0.8_N barrier layer (n~1 × 10^15^ cm^−3^), and a 300-nm-thick GaN channel layer (n~1 × 10^15^ cm^−3^) from top to bottom. The gate length (*L*_g_) and gate width (*W*_g_) of the device are 4 µm and 100 µm, respectively. The gate-source distance (*L*_gs_) and gate-drain distance (*L*_gd_) are 2 µm. The operating principle of the device was illustrated in our previous work [17].

Steady-state 2-D numerical simulations based on Silvaco TCAD Atlas software are performed. The definition of fundamental equations and physical models can be found in our previous work [18]. The spaces between the gate source and gate drain are filled with SiO_2_. The source and drain electrodes are defined as ohmic contacts. The wavelength and intensity of the incident light are set to 360 nm and 1 mW/cm^2^, respectively.

## 3. Results and Discussion

### 3.1. Hole Concentration of the p-GaN Layer

**Figure 2:**
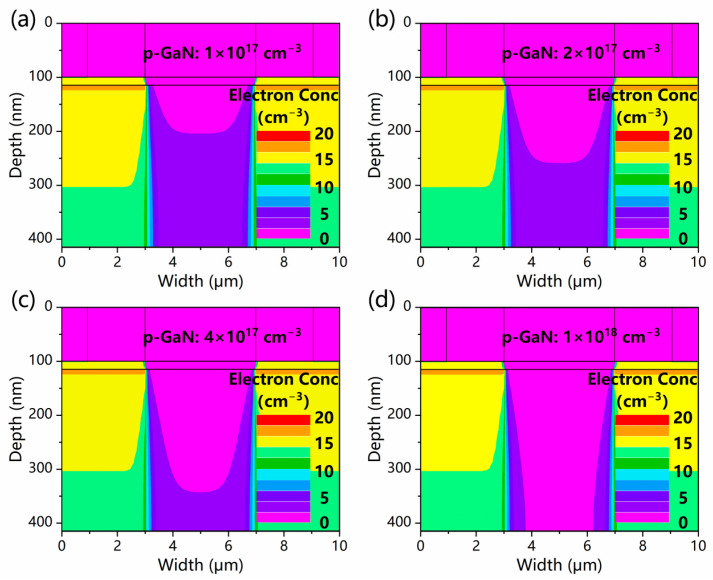
The electron concentrations of the GaN channel layers with various p-GaN hole concentrations of (**a**) 1 × 10^17^ cm^−3^, (**b**) 2 × 10^17^ cm^−3^, (**c**) 4 × 10^17^ cm^−3^, and (**d**) 1 × 10^18^ cm^−3^.

The simulation results show that a higher hole concentration in the p-GaN layer leads to a deeper depletion region in the GaN channel layer, as shown in Figure 2. For a 300 nm GaN layer that can completely absorb the incident light, a hole concentration of 1 × 10^18^ cm^−3^ in the p-GaN layer is essential to fully deplete the GaN layer and suppress the leakage current. Because it is difficult to further increase the hole concentration of the p-GaN layer in MOCVD growth [19], the value is determined to be 1 × 10^18^ cm^−3^ in later simulations.

### 3.2. Thickness of the p-GaN Layer

**Figure 3:**
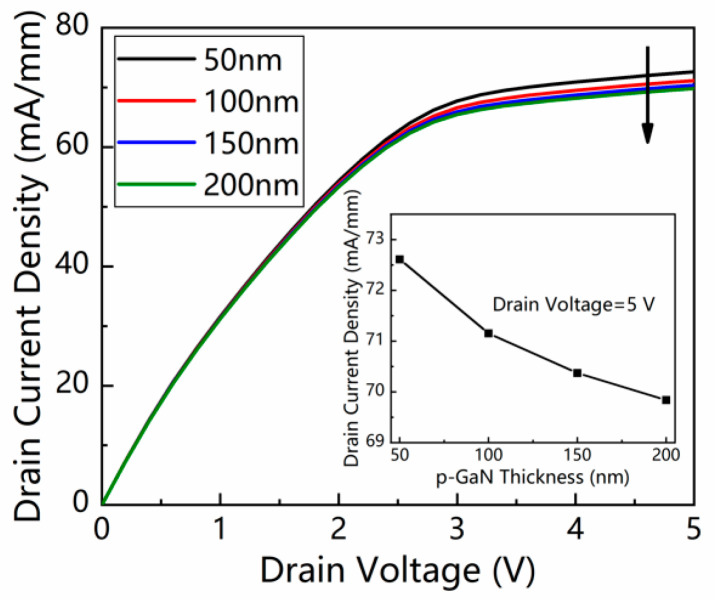
*I*–*V* characteristics of the device under illumination with various p-GaN layer thicknesses. The light intensity was 1 mW/cm^2^. The insert shows the drain current density as a function of the p-GaN thickness from 50 to 200 nm at a drain voltage of 5 V.

With the increase in the p-GaN layer thickness from 50 to 200 nm, the photocurrent density between the source and drain decreases monotonically from 72.61 to 69.84 mA/mm, as presented in Figure 3. The reduction of photocurrent is due to the absorption loss of the incident light in the p-GaN layer. As a result, a thinner p-GaN is preferred to maintain a high photocurrent. However, a thickness of approximately 50 nm is essential to ensure the material quality and doping stability of p-GaN during MOCVD growth [20]. As a consequence, a trade-off should be made, and the thickness of the p-GaN layer is selected to be 50 nm in later simulations.

### 3.3. Thickness of the GaN Channel Layer

**Figure 4:**
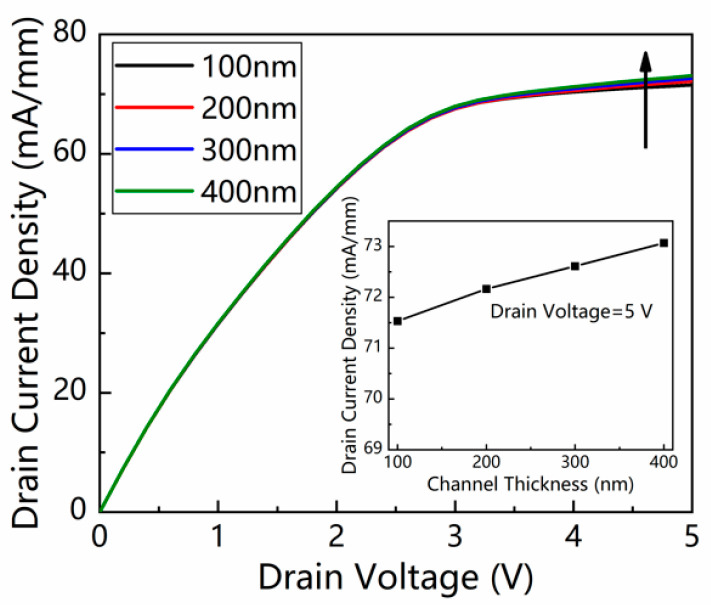
*I*–*V* characteristics of the device under illumination with various GaN channel layer thicknesses. The light intensity was 1 mW/cm^2^. The insert is the drain current density as a function of GaN channel thickness from 100 to 400 nm at 5 V.

With the increase in the GaN channel layer thickness from 100 to 400 nm, the photocurrent density between the source and drain rises slightly from 71.53 to 73.07 mA/mm, as presented in Figure 4. The increase of photocurrent is due to the extended absorption depth of the incident light in the GaN channel layer. As a result, a thicker GaN channel is preferred to maintain a high photocurrent.

**Figure 5:**
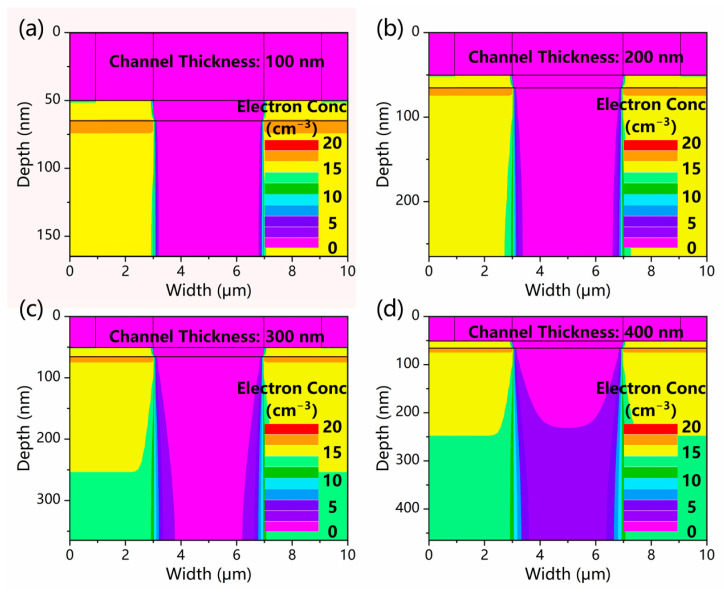
The electron concentration distributions of the GaN channel in dark conditions with various GaN channel thicknesses of (**a**) 100 nm, (**b**) 200 nm, (**c**) 300 nm, and (**d**) 400 nm.

However, as the thickness exceeds 300 nm, the GaN layer cannot be fully depleted, as displayed in Figure 5d. This will result in increased leakage current and degenerated device performance, as discussed before. As a consequence, a trade-off should also be made, and the thickness of the GaN channel layer is selected to be 300 nm in later simulations.

### 3.4. Al Content and Thickness of the AlGaN Barrier Layer

**Figure 6:**
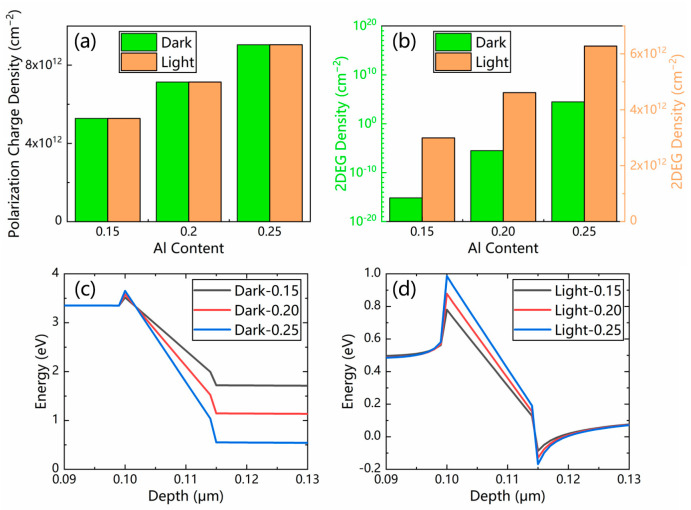
(**a**) Polarization charge density and (**b**) 2DEG density of the p-GaN/AlGaN/GaN PTs with three different values of Al molar fraction. The simulated conduction band diagrams (**c**) in the dark and (**d**) under illumination. The 2DEG densities in the dark and under illumination are displayed in two different columns in Figure 6b for a clear observation.

Figure 6a demonstrates the polarization charge density at the AlGaN/GaN heterojunction interface, which is induced by spontaneous and piezoelectric polarization. In dark conditions, the polarization-induced 2DEG is depleted by the p-GaN layer, as presented in Figure 6b. Additionally, the conduction band of the GaN channel is flattened, as illustrated in Figure 6c. Under UV illumination, no obvious difference was observed for the polarization charge density, while the 2DEG was restored, as shown in Figure 6a and Figure 6b, respectively. The conduction band sinks at the heterojunction interface, as illustrated in Figure 6d.

The Al content and thickness of the AlGaN barrier layer are two critical parameters adjusting the 2DEG density, which determines the magnitude of the photocurrent. With an increase in the Al content from 0.15 to 0.25, both the spontaneous polarization and piezoelectric polarization are continuously enhanced. As a result, the increased Al content of the AlGaN barrier layer leads to a larger polarization charge density, a higher 2DEG density, and a bigger conduction band offset, as presented in Figure 6.

**Figure 7:**
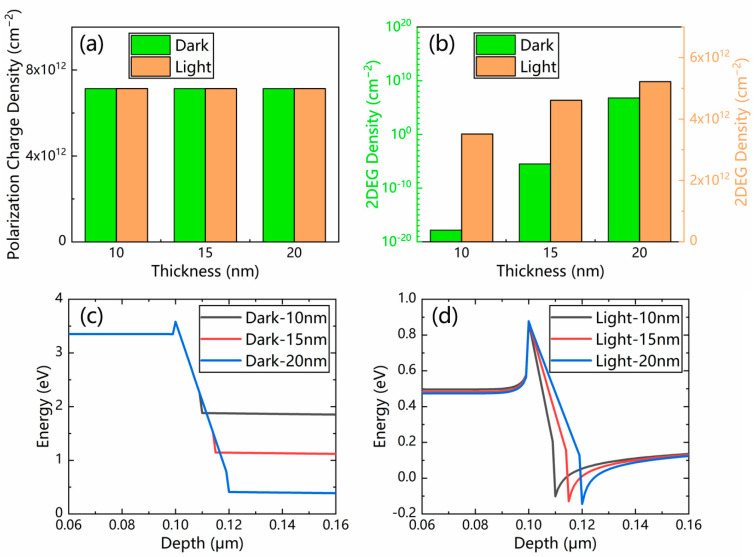
(**a**) Polarization charge density and (**b**) 2DEG density of the p-GaN/AlGaN/GaN PTs with three different values of AlGaN layer thickness. The simulated conduction band diagrams (**c**) in the dark and (**d**) under illumination.

With the increase in the AlGaN layer thickness from 10 to 20 nm, the polarization charge density remains the same, as presented in Figure 7a. However, a higher 2DEG density is observed, both in the dark and under illumination, as presented in Figure 7b. The increased thickness of the AlGaN layer also leads to a bigger conduction band offset, as presented in Figure 7c,d. As a result, a larger photocurrent can be achieved with a thicker AlGaN layer.

**Figure 8:**
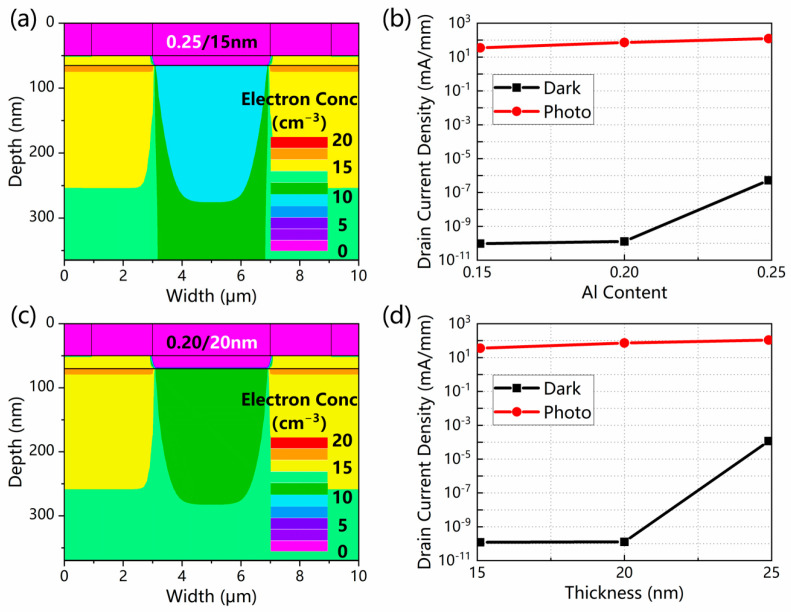
The electron concentration distributions of the GaN channel in dark conditions for the structure of (**a**) a 15-nm-thick AlGaN barrier layer with an Al content of 0.25 and (**c**) a 20-nm-thick AlGaN barrier layer with an Al content of 0.20. The drain current density as a function of (**b**) Al content and (**d**) thickness of the AlGaN layer at 5 V.

It is worth noting that despite the improved photocurrent, a larger Al content or greater thickness for the AlGaN layer also leads to an increased leakage current. With a larger Al content of 0.25, the 300 nm GaN channel layer could not be completely depleted, and the leakage current density rose significantly, as displayed in Figure 8a and Figure 8b, respectively. With a greater AlGaN layer thickness of 20 nm, similar conditions are shown in Figure 8c,d. As a result, optimizations should be made on the Al content and thickness of the AlGaN layer.

**Figure 9:**
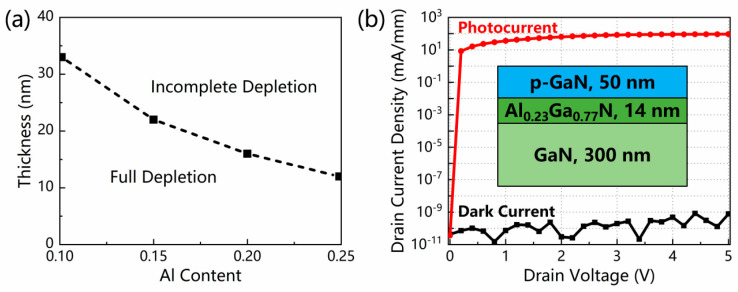
(**a**) The depletion conditions of the GaN channel layer with various Al contents and thicknesses of the AlGaN layer. The critical thicknesses for Al contents of 0.10, 0.15, 0.20, and 0.25 are 33, 22, 16, and 12 nm, respectively. (**b**) *I*–*V* characteristics of the optimized device in the dark and under illumination with an Al content of 0.23 and a thickness of 14 nm. The light intensity was 1 mW/cm^2^. The insert of Figure 9b is a schematic of the optimized device structure.

Figure 9a plots the depletion conditions of the GaN channel layer with various Al contents and thicknesses of the AlGaN layer. The critical thicknesses of full depletion at Al contents of 0.10, 0.15, 0.20, and 0.25 are 33, 22, 16, and 12 nm, respectively, as marked by the dotted line. To ensure the depletion of the GaN layer, the values of the Al content and thickness of the AlGaN layer should be located in the lower left of the borderline, which is similar to that in [21].

Under this premise, more detailed simulations are conducted with Al content in 0.01 steps and thickness in 1 nm steps. The results reveal that the device with an Al content of 0.23 and a thickness of 14 nm has the highest photocurrent density of 92.11 mA/mm while maintaining a low dark current density of 7.68 × 10^−10^ mA/mm and a large photo-to-dark-current ratio of over 10^11^ at a drain voltage of 5 V, as presented in Figure 9b. As a consequence, the optimized structure is determined, as shown in the insert of Figure 9b. Compared with our previous work [17], the optimized structure demonstrates a similar photocurrent density and photo-to-dark-current ratio with a light intensity that is one magnitude lower.

## 4. Conclusions

In summary, a comprehensive simulation via Silvaco Atlas was conducted to reveal the impact of the structural parameters on the performances of p-GaN/AlGaN/GaN HEMT-based UV PTs. The hole concentration and thickness of the p-GaN layer as well as the thickness of the GaN channel layer are studied and optimized. The depletion conditions with various Al contents and thicknesses of the AlGaN barrier layer are investigated in detail, and a borderline between full depletion and incomplete depletion was drawn. Finally, an optimized structure with an Al content of 0.23 and a thickness of 14 nm is achieved for UV-PT, which exhibits the highest photocurrent density of 92.11 mA/mm, a low dark current density of 7.68 × 10^−10^ mA/mm, and a large photo-to-dark-current ratio of over 10^11^ at a drain voltage of 5 V. We believe that the results have drawn a clear physical map of HEMT-based PTs and could be a useful guide for device design for subsequent researchers.

## Data Availability

Not applicable.
